# On Sample Size Determination for Augmented Tests Based on Restricted Mean Survival Time in Randomized Clinical Trials

**DOI:** 10.1002/bimj.70046

**Published:** 2025-03-24

**Authors:** Satoshi Hattori, Hajime Uno

**Affiliations:** ^1^ Department of Biomedical Statistics Graduate School of Medicine and Integrated Frontier Research for Medical Science Division Institute for Open and Transdisciplinary Research Initiatives (OTRI) Osaka University Suita City Osaka Japan; ^2^ Department of Medical Oncology Division of Population Sciences Dana‐Farber Cancer Institute Department of Medicine Harvard Medical School Massachusetts USA

**Keywords:** augmentation, blinded sample size re‐estimation, martingale residual, nonproportional hazards

## Abstract

Restricted mean survival time (RMST) is gaining attention as a measure to quantify the treatment effect on survival outcomes in randomized clinical trials. Several methods to determine sample size based on the RMST‐based tests have been proposed. However, to the best of our knowledge, there is no discussion about the power and sample size regarding the augmented version of RMST‐based tests, which utilize baseline covariates for a gain in estimation efficiency and in power for testing no treatment effect. The conventional event‐driven study design based on the logrank test allows us to calculate the power for a given hazard ratio without specifying the survival functions. In contrast, the existing sample size determination methods for the RMST‐based tests relies on the adequacy of the assumptions of the entire survival curves of two groups. Furthermore, to handle the augmented test, the correlation between the baseline covariates and the martingale residuals must be handled. To address these issues, we propose an approximated sample size formula for the augmented version of the RMST‐based test, which does not require specifying the entire survival curve in the treatment group, and also a sample size recalculation approach to update the correlations between the baseline covariates and the martingale residuals with the blinded data. The proposed procedure will enable the studies to have the target power for a given RMST difference even when correct survival functions cannot be specified at the design stage.

## 1 Introduction

In randomized clinical trials designed to compare two treatments with a time‐to‐event outcome, the logrank test is extensively used for testing equality of the two event time distributions. To summarize the treatment effect magnitude, the hazard ratio (HR) is widely used, which is estimated with the Cox proportional hazards (PH) model (Cox 1972). The logrank test and the HR are used together and in this paper, this approach is referred to as the logrank‐HR approach. On the other hand, the PH assumption the Cox PH model requires is not necessarily satisfied in practice. Concerning the inappropriateness of the PH assumption, inference procedures for many kinds of semiparametric non‐PH models have been developed, including the accelerated failure time model (Jin et al. 2003; Wei 1992), the proportional odds model (Cheng et al. 1995), and the additive hazards model (Lin and Ying 1994). These inference procedures were found to perform well in some practical situations. On the other hand, these semiparametric models also rely on some specific assumptions regarding the relationship between two event time distributions, such as additive hazards or proportional odds assumptions, and then are also subject to misspecification similar to the Cox PH model. They have been rarely employed in confirmatory randomized clinical trials; instead the logrank‐HR approach has been routinely used (Uno et al. 2020).

Many recent clinical trials of immunotherapies for cancer reported that the Kaplan–Meier curves of the two treatment arms are almost identical up to a certain time point from the randomization and after the time point, the two curves separated, indicating that the immunotherapy improved patients' survival (Guimaraes et al. 2020; Reck et al. 2016). This late‐onset efficacy reflects the mechanism of the immunotherapy; a certain duration is needed for the immunotherapy to act on the immune system. In this case, violation of the PH assumption is essential from the viewpoint of the mechanism of the therapy. It motivates statisticians to consider more closely how to analyze the primary time‐to‐event endpoint in confirmatory randomized clinical trials without assuming the PH assumption (non‐PH). Uno et al. (2014) contrasted the pros and cons of several measures for the treatment effect alternative to the HR, including difference and ratio of the restricted mean survival time (RMST), which is the mean survival time truncated at a specific study time and calculated as the area under the survival curve from 0 to the truncation time point (Royston and Parmar 2011;2013; Tian et al. 2018; Uno et al. 2014;2015). Difference and ratio of RMSTs of the two groups can be good between‐group contrast measures with clear clinical interpretation. These measures can be estimated nonparametrically without imposing any modeling assumptions, and thus are robust. This model‐free property would be very attractive in confirmatory randomized clinical trials because the statistical analysis specified in the study protocol (CPMP 2003) would always give the interpretation intended in the study protocol; the HR does not have this property once the PH assumption is violated. With the awareness of the issues of the HR, the RMST is gaining more attention in the clinical research community and is starting to be utilized in practice. A study where the RMST‐based analysis was used as the primary analysis can also be found (Guimaraes et al. 2020).

Although our primary focus is on the augmented tests based on the RMST, in which efficacy and power are improved with baseline covariates, we begin with the standard tests; we refer to the test and estimate without covariates incorporated to improve efficiency and power simply as the standard test and estimate, respectively. There are several methods for calculating sample sizes available for the standard tests contrasting the RMST difference between two comparative groups. Royston and Parmar (2013) showed a simulation‐based method to determine the sample size for RMST. Uno et al. (2015) discussed a simulation‐based method specifically for noninferiority trials. Luo et al. (2019) and Eaton et al. (2020) discussed the use of an asymptotic power formula. These power calculation approaches require users to specify the entire survival curves of the two groups, and some simple parametric distributions have been assumed conventionally. For example, in Guimaraes et al. (2020), the exponential distribution was assumed and the rate parameter of the exponential distribution was determined so that the 1‐year survival rate became 0.855. If another parametric model, for example, a log‐normal model, was used for the power calculation, the resulting power would be different from the one based on the exponential distribution even if the 1‐year survival rate was 0.855. For estimating the sample size more accurately, one may assume piecewise exponential distributions instead of simple parametric distributions as Luo et al. (2019) proposed. However, in practice, it would be still challenging to accurately specify the entire survival curves at the design stage due to limited information about the treatment. If the specified survival curves are inaccurate, the sample size based on RMST‐based tests may be under/overestimated because the power formula involves the entire survival curves of two groups (Eaton et al. 2020; Luo et al. 2019).

On the other hand, the power formula for the logrank test, or equivalent HR‐based tests (Schoenfeld et al. 1981) does not involve the entire survival curves but only a required number of events, anticipated HR, and type 1 error rate. Thus, when the logrank test is used as the primary analysis, the final analysis is supposed to be conducted when the required number of events is observed to achieve the planned power. This is called an “event‐driven study” design and has been almost routinely used for decades (Collett 2004). This approach still requires users to specify the entire survival curves of two groups, the anticipated accrual profile, and the follow‐up duration to calculate the total number of subjects to enroll. Misspecification of the survival curves may lead to unexpected delays in the final analysis. However, because the final analysis is performed when the required number of events is observed and the power of the logrank test depends on only the number of observed events, misspecification of the survival curves would not affect the power of the study. This is a practical advantage of using the logrank test against RMST‐based tests and weighted logrank tests (Yuan et al. 2020).

The potential usefulness of baseline covariates in the primary statistical analysis has been argued for a long time (DiRienzo and Lagakos 2001; Pocock et al. 2002; Tsiatis et al. 1985). However, in most clinical trials, no or only a few baseline covariates are incorporated in the primary analysis; due to potential misspecification, regression analyses are hardly used and only limited number of covariates are adjusted with stratified analysis. The augmentation approach is gaining much interest, in which covariates are used to reduce variations of estimators by attaching an augmented term to the estimators or the estimating equations (Hattori et al. 2022; Jiang et al. 2019; Lu and Tsiatis 2008; Tian et al. 2012; Tsiatis et al. 2008; Zhang 2015; Zhang et al. 2008). With randomization, the addition of the augmented term does not lead any bias to estimators and does not require any additional assumptions for validity. For the RMST‐based tests, Tian et al. (2012) and Jiang et al. (2019) discussed the inference procedure. However, to the best of our knowledge, there has been no discussion about power and sample size calculations for the RMST‐based tests with the augmentation.

In this paper, we sought to develop a procedure that does not require a correct specification of survival functions and censoring distributions at the design stage but can allow the study to achieve a target power for detecting a given RMST difference. We handle this problem under the same assumptions as the event‐driven design with the logrank test; although the common censoring assumption between the two groups is not required for the analysis, it is supposed to obtain a simple procedure for sample size calculation. We derive the asymptotic power formula under a local alternative, which is useful in a blinded review. For the standard test, with this formula, given the target type 1 error rate and the power, the sample size is determined by an anticipated difference in RMST, the survival distribution of the control group, and censoring distribution. For the augmented test, in addition to these quantities, a correlation among baseline covariates and the martingale residuals should be set. To determine the quantities needed for the sample size calculation, one may utilize data from existing clinical data sets, such as past clinical trials. We refer to such a data set as the *reference data*, whereas the study we are designing is called the *target study*. This approach can be taken at the design stage. If the quantities determined through the *reference data* are similar to those for the *target study*, the power based on the proposed formula will be accurate for the target study. Otherwise, it will not be accurate. Therefore, along with the power formula, we also propose a method to recalculate the sample size in the middle of the study with the blinded data. Since this sample size calculation is performed without breaking the blindness of the assigned treatment, the integrity of the study will be intact and the impact on the type I error rate will be negligible.

The organization of the paper is as follows. Although our development covers the augmented RMST‐based test, our consideration on sample size calculation would be useful even with the standard RMST‐based test. In Section 2.1, we begin with summarizing the asymptotic properties of the standard test for the RMST difference, and in Section 2.2, introduce the augmented RMST‐based test. In Section 3, we derive the asymptotic power formula under a local alternative. In Section 4.1, we demonstrate the sample size calculation at the design‐stage with the power formula for the standard test and the augmented tests. It is followed by a proposal of a mid‐trial sample size modification procedure in Section 4.2. In Section 5, we report results of a simulation study. In Section 6, we demonstrate the application of the proposed methods to real data. We conclude our paper by mentioning some limitations and the potential future direction of the research in Section 7. All the theoretical arguments are given in the Appendix.

## 2 RMST‐Based Tests

### 2.1 The Standard Test Based on the RMST Difference

Suppose we are interested in designing a randomized clinical trial with a time‐to‐event endpoint. We call the clinical trial the *target study*. We consider a two group comparison and let Z be a binary random variable with P(Z=1)=π, which represents the treatment allocation and is coded as 1 and 0 if a subject is allocated to the treatment and control groups, respectively. We only consider equal allocation cases; π = 0.5. Let T and C be a failure time of interest and potential censoring time, respectively. The failure time T may be right‐censored by C and then X=min(T,C) and Δ=I(T≤C) are observable. A vector of baseline covariates is denoted by V. From randomization, we assume

$$\begin{array}{c} \mathrm{Condition}1:\;\;V⊥Z, \end{array}$$

where for arbitrary random variables $A_{1}$ and $A_{2}$, $A_{1}⊥A_{2}$ implies independence of $A_{1}$ and $A_{2}$. In addition, we assume the standard assumption in survival analysis;

$$\begin{array}{c} \mathrm{Condition}2:\;\;C⊥T|Z, \end{array}$$

where for arbitrary random variables $A_{1},A_{2}$, and $A_{3}$, $A_{1}⊥A_{2}|A_{3}$ implies that $A_{1}$ and $A_{2}$ are conditionally independent given $A_{3}$.

We assume that n subjects are enrolled in the study. Let n i.i.d. copies of $\left(X,\Delta ,Z,V^{T}\right)$ denoted by $(X_{i},\Delta_{i},Z_{i},V_{i}^{T}),i=1,2,\text{…},n$ and observed, where the subscript i represents the ith subject. Let $S_{z}\left(t\right)=P\left(T\geq t|Z=z\right)$ be the survival function of the group z=0,1. Denote the corresponding hazards and cumulative hazards function by $\lambda_{z}\left(t\right)$ and $\Lambda_{z}\left(t\right)$, respectively. The counting process and the at‐risk process are denoted by $N_{i}\left(t\right)=I\left(X_{i}\leq t,\Delta_{i}=1\right)$ and $Y_{i}\left(t\right)=I\left(X_{i}\geq t\right)$, respectively. The RMST over the interval [0,τ] for Z=z is defined as $\theta_{z}=E\left\{min\left(T,\tau \right)|Z=z\right\}=\int_{0}^{\tau }S_{z}\left(t\right)dt$, where τ is a truncation time. Suppose we employ the RMST to summarize the treatment effect and the truncation time τ is predefined in the protocol. The RMST is estimated by ${\hat{\theta }}_{z}=\int_{0}^{\tau }{\hat{S}}_{z}\left(t\right)dt$, where ${\hat{S}}_{z}\left(t\right)$ is the Kaplan–Meier estimator for $S_{z}\left(t\right)$. To compare the two treatments, the RMST difference can be used, which is defined by $\theta =\theta_{1}-\theta_{0}$. It is estimated by $\hat{\theta }={\hat{\theta }}_{1}-{\hat{\theta }}_{0}$. As shown in Appendix A, the asymptotic variance of $\sqrt{n}\left(\hat{\theta }-\theta \right)$ is given by

(1) $$\begin{array}{cc} \sigma^{2} & =\int_{0}^{\tau }\frac{{\left\{\int_{t}^{\tau }S_{1}\left(u\right)du\right\}}^{2}}{E\{I(X\geq t)Z\}}d\Lambda_{1}\left(t\right)+\int_{0}^{\tau }\frac{{\left\{\int_{t}^{\tau }S_{0}\left(u\right)du\right\}}^{2}}{E\{I(X\geq t)(1-Z)\}}d\Lambda_{0}\left(t\right). \end{array}$$

By replacing the unknown quantities in (1) with their consistent estimators, it is consistently estimated by

(2) $$\begin{array}{cc} {\hat{\sigma }}_{1}^{2} & =\int_{0}^{\tau }\frac{{\left\{\int_{t}^{\tau }{\hat{S}}_{1}\left(u\right)du\right\}}^{2}}{{\bar{Y}}_{1}\left(t\right)}d{\hat{\Lambda }}_{1}\left(t\right)+\int_{0}^{\tau }\frac{{\left\{\int_{t}^{\tau }{\hat{S}}_{0}\left(u\right)du\right\}}^{2}}{{\bar{Y}}_{0}\left(t\right)}d{\hat{\Lambda }}_{0}\left(t\right), \end{array}$$

where ${\bar{Y}}_{1}\left(t\right)=n^{-1}\sum_{i=1}^{n}I\left(X_{i}\geq t\right)Z_{i}$, ${\bar{Y}}_{0}\left(t\right)=n^{-1}\sum_{i=1}^{n}I\left(X_{i}\geq t\right)\left(1-Z_{i}\right)$, and ${\hat{\Lambda }}_{z}\left(t\right)=\int_{0}^{t}\sum_{i=1}^{n}I\left(Z_{i}=z\right){\left\{n{\bar{Y}}_{z}\left(u\right)\right\}}^{-1}dN_{i}\left(u\right)$ is the Nelson–Aalen estimate for $\Lambda_{z}\left(t\right)$. Alternatively, $\sigma^{2}$ is also consistently estimated by ${\hat{\sigma }}_{2}^{2}=n^{-1}\sum_{i=1}^{n}{\hat{H}}_{i}^{2}$, where

(3) $$\begin{array}{cc} {\hat{H}}_{i} & =-\int_{0}^{\tau }\frac{\int_{t}^{\tau }{\hat{S}}_{1}\left(u\right)du}{{\bar{Y}}_{1}\left(t\right)}Z_{i}d{\hat{M}}_{1,i}\left(t\right)\\ & \;+\int_{0}^{\tau }\frac{\int_{t}^{\tau }{\hat{S}}_{0}\left(u\right)du}{{\bar{Y}}_{0}\left(t\right)}\left(1-Z_{i}\right)d{\hat{M}}_{0,i}\left(t\right), \end{array}$$

where ${\hat{M}}_{z,i}\left(t\right)=N_{i}\left(t\right)-\int_{0}^{t}I\left(X_{i}\geq u\right)d{\hat{\Lambda }}_{z}\left(u\right)$ is the counting process martingale for Z=z. The consistency of ${\hat{\sigma }}_{2}^{2}$ to ${\hat{\sigma }}^{2}$ is given in Appendix A. We refer the test based on $\hat{\theta }$ as the standard RMST test.

### 2.2 Augmented Test for the RMST Difference

The augmented version of the RMST difference is defined as

(4) $$\begin{array}{ccc} {\hat{\theta }}_{aug}\left(c\right) & = & \hat{\theta }-\frac{1}{n}\sum_{i=1}^{n}\left(Z_{i}-\pi \right)c^{T}V_{i},\\ & = & \hat{\theta }-AUG\left(c\right), \end{array}$$

where c is a vector of the same dimension as V (Tian et al. 2012). As argued in Section 2.1, the first term of (4) consistently estimates the true RMST difference θ. For any c, the expectation of the second term is zero from Condition 1. Then (4) consistently estimates the true RMST difference for any fixed c. It is true even if c is date‐dependent as long as it converges to a constant in probability. Since $\hat{\theta }$ is a special case of ${\hat{\theta }}_{aug}\left(c\right)$ with c= 0, by choosing a relevant value of c, one may have a more efficient estimator than $\hat{\theta }$. We determine c that minimizes the variance. The resulting minimizer is denoted by $\hat{c}$, which can be obtained by projecting the influence function of $\hat{\theta }$ onto the subspace of $L^{2}\left(dP\right)$ spanned by {(Z−π)V}, where dP is the probability measure of the underlying probability space and $L^{2}\left(dP\right)$ is the Hilbert space of all the square‐integrable functions on the probability space. As argued in Appendix B, it is given by

(5) $$\begin{array}{cc} \hat{c} & ={\left\{\pi \left(1-\pi \right)\sum_{i=1}^{n}V_{i}V_{i}^{T}\right\}}^{-1}\\ & \;\times \sum_{i=1}^{n}\left(Z_{i}-\pi \right)V_{i}[-Z_{i}\int_{0}^{\tau }\frac{\int_{t}^{\tau }{\hat{S}}_{1}\left(u\right)du}{{\bar{Y}}_{1}\left(t\right)}d{\hat{M}}_{1i}\left(t\right)\\ & \;+\left(1-Z_{i}\right)\int_{0}^{\tau }\frac{\int_{t}^{\tau }{\hat{S}}_{0}\left(u\right)du}{{\bar{Y}}_{0}\left(t\right)}d{\hat{M}}_{0i}\left(t\right)]. \end{array}$$

Let ${\hat{\theta }}_{aug}={\hat{\theta }}_{aug}\left(\hat{c}\right)$. Then, θ is estimated by ${\hat{\theta }}_{aug}$ consistently and more efficiently than $\hat{\theta }$. The asymptotic variance of $\sqrt{n}\left({\hat{\theta }}_{aug}-\theta \right)$ is consistently estimated by ${\hat{\sigma }}_{aug}^{2}=n^{-1}\sum_{i=1}^{n}{\left\{{\hat{H}}_{i}-\left(Z_{i}-\pi \right){\hat{c}}^{T}V_{i}\right\}}^{2}$ (see Appendix B).

## 3 Power Formula for the Standard and Augmented RMST Tests

To obtain a simple expression of the local power, we assume an additional condition,

$$\begin{array}{c} \mathrm{Condition}3:\;\;C⊥Z. \end{array}$$

This condition is assumed in the widely used power formula for the event‐driven study by the logrank test (see exercise 4.7 of Fleming and Harrington 1991). Then, the variance (1) is represented as

(6) $$\begin{array}{cc} \sigma^{2} & =\int_{0}^{\tau }\frac{{\left\{\int_{t}^{\tau }S_{1}\left(u\right)du\right\}}^{2}}{\pi S_{1}\left(t\right)G\left(t\right)}d\Lambda_{1}\left(t\right)+\int_{0}^{\tau }\frac{{\left\{\int_{t}^{\tau }S_{0}\left(u\right)du\right\}}^{2}}{\left(1-\pi \right)S_{0}\left(t\right)G\left(t\right)}d\Lambda_{0}\left(t\right), \end{array}$$

where G(t)=P(C>t) is the common survival function of the censoring time C. Since $\hat{c}$ is derived by the orthogonal projection of the influence function, it holds that

$$\begin{array}{cc} \lim_{n\to \infty }Var\left(\sqrt{n}\left({\hat{\theta }}_{aug}-\theta \right)\right) & =\lim_{n\to \infty }Var\left(\sqrt{n}\left(\hat{\theta }-\theta \right)\right)\\ & \;-\lim_{n\to \infty }Var\left(\sqrt{n}AUG\left(\hat{c}\right)\right)\\ & =Q_{1}-Q_{2}. \end{array}$$

Note that $Q_{1}$ agrees with $\sigma^{2}$ in (6). Suppose we are interested in testing the null hypothesis that the survival functions are common between the groups. It is denoted by $H_{0}:\log\lambda_{1}\left(t\right)/\lambda_{0}\left(t\right)=0$. We consider the local alternative $H_{1}:\log\lambda_{1}\left(t\right)/\lambda_{0}\left(t\right)=\delta \left(t\right)/\sqrt{n}$, where δ(t) is a deterministic function of time providing a specific alternative hypothesis of interest.

Under this alternative, $\sqrt{n}\left(\hat{\theta }-\theta_{alt}\right)$ asymptotically has a zero‐mean normal distribution with variance $\sigma^{2}$ in Equation (6), where

$$\begin{array}{c} \theta_{alt}=\frac{\eta }{\sqrt{n}}=\frac{1}{\sqrt{n}}\int_{0}^{\tau }\left\{\int_{0}^{v}\delta \left(u\right)\lambda_{0}\left(u\right)du\right\}S_{0}\left(v\right)dv. \end{array}$$

Under the local alternative and Conditions 2 and 3, it holds that $S_{1}\left(t\right)=S_{0}\left(t\right)+o\left(1\right)$, $\Lambda_{1}\left(t\right)=\Lambda_{0}\left(t\right)+o\left(1\right)$, $M_{1,i}\left(t\right)=M_{0,i}\left(t\right)+o_{p}\left(1\right)$, $E\left\{I\left(X\geq t\right)Z\right\}=S_{1}\left(t\right)G\left(t\right)\pi =S_{0}\left(t\right)G\left(t\right)\pi +o\left(1\right)$, and $E\left\{I\left(X\geq t\right)\left(1-Z\right)\right\}=S_{0}\left(t\right)G\left(t\right)\left(1-\pi \right)$. Applying these identities to (6), we can approximate $Q_{1}$ by $Q_{1}=\sigma^{2}={\tilde{\sigma_{1}}}^{2}+o_{p}\left(1\right)$, where

(7) $$\begin{array}{cc} {\tilde{\sigma }}_{1}^{2} & =\frac{1}{\pi (1-\pi )}\int_{0}^{\tau }\frac{{\left\{\int_{t}^{\tau }S_{0}\left(u\right)du\right\}}^{2}}{S_{0}\left(t\right)G\left(t\right)}d\Lambda_{0}\left(t\right), \end{array}$$

and then from the Slutsky's theorem (Ferguson 1996), it holds that $\sqrt{n}\left(\hat{\theta }-\theta_{alt}\right)$ asymptotically follows $N(0,{\tilde{\sigma_{1}}}^{2})$.

In Appendix C, we show that when π=1/2, it holds that

(8) $$\begin{array}{c} Q_{2}=\pi \left(1-\pi \right)e_{2}, \end{array}$$

where

(9) $$\begin{array}{ccc} e_{2} & = & E\left\{\int_{0}^{\tau }\frac{\int_{t}^{\tau }S_{0}\left(u\right)du}{S_{0}\left(t\right)G\left(t\right)}dM_{0}\left(t\right)V^{T}\right\}{\left\{E\left(VV^{T}\right)\right\}}^{-1}\\ & & \times E\left\{\int_{0}^{\tau }\frac{\int_{t}^{\tau }S_{0}\left(u\right)du}{S_{0}\left(t\right)G\left(t\right)}dM_{0}\left(t\right)V\right\}, \end{array}$$

and $M_{0}\left(t\right)=N\left(t\right)-\int_{0}^{\tau }Y\left(u\right)d\Lambda_{0}\left(u\right)$ is the martingale residuals under the null hypothesis. Thus, the variance of ${\hat{\theta }}_{aug}$ is asymptotically approximated by $v_{aug}^{2}=\left\{{\tilde{\sigma }}^{2}-\pi \left(1-\pi \right)e_{2}\right\}/n$. Then, the local power for a two‐sided α level test is given by

(10) $$\begin{array}{c} \Phi \left(z_{\alpha /2}-\theta_{alt}/v_{aug}\right)+1-\Phi \left(z_{\left(1-\alpha /2\right)}-\theta_{alt}/v_{aug}\right). \end{array}$$

If one uses the standard test, which is based on $\hat{\theta }$, the power is approximately calculated by setting $e_{2}=0$, or

(11) $$\begin{array}{c} \Phi \left(z_{\alpha /2}-\theta_{alt}/v\right)+1-\Phi \left(z_{\left(1-\alpha /2\right)}-\theta_{alt}/v\right), \end{array}$$

where $v^{2}={\tilde{\sigma }}^{2}/n$.

Note that these power formulas, (10) and (11), are derived based on the local alternative hypothesis. These might not provide precise approximation of the power when the treatment effect is large. However, this approximated approach is more convenient than the one using (1) for practice. For example, for the power calculation of the standard test, we need to specify the entire survival curves from the two groups ($S_{1}\left(t\right)$ and $S_{0}\left(t\right)$), and G(t), when we use the approach based on $\sigma^{2}$. On the other hand, the approximated approach using ${\tilde{\sigma }}_{1}^{2}$ requires us to specify only a between‐group difference in RMST (η), G(t), and the entire survival curve from the control group $\left(S_{0}\left(t\right)\right)$. This feature would be attractive to users since they would not have sufficient data to estimate the survival time distribution, especially in the treatment group at the study design stage.

## 4 Sample Size Calculation

### 4.1 Sizing at the Design Stage

In this subsection, we discuss sample size calculation for a randomized clinical trial with the RMST‐based test at the design stage. We begin with the case in which the standard RMST‐based test is used for the primary analysis with two‐tailed significance level of 0.05. We define the target sample size to maintain the target power 1−β for the minimum clinically meaningful difference $\theta_{alt}$. As given in (11), the power depends on the survival function of the control group $S_{0}\left(t\right)$ and the censoring distribution G(t). To accurately estimate the sample size achieving the target power, one needs to carefully specify $S_{0}\left(t\right)$ and G(t) with available information at the design stage. Suppose we are planning a *target study* with the *reference data*, for example, from past clinical trials. When information for the control group is available, from the definition of ${\tilde{\sigma }}_{1}^{2}$ in (7), one can approximately calculate the power by estimating $S_{0}\left(t\right)$ and G(t) with the Kaplan–Meier method. If (almost) all the subjects are not dropped out from the study, the censoring is administrative and G(t) can be determined by the design parameters such as accrual rate and accrual period. It can be used instead of estimation.

Next, we consider the case in which the augmented RMST test is used. To do so, we need to estimate $e_{2}$. It depends on the martingale residuals under the null, which is free from the treatment allocation Z. Then, one can estimate $v_{aug}^{2}$ with a data set of only the control group. Suppose we have $n_{+}$ subjects in the *reference data* and the same notation to Section 2 is used. Note that Z=0 for all the subjects. Then, the predicted power is obtained by replacing unknown quantities in (9). That is, it can be estimated by

$$\begin{array}{ccc} {\hat{e}}_{2} & = & \frac{1}{n_{+}}\sum_{i=1}^{n_{+}}\int_{0}^{\tau }\frac{\int_{t}^{\tau }{\hat{S}}_{0}\left(u\right)du}{{\bar{Y}}_{0}\left(t\right)}d{\hat{M}}_{0,i}\left(t\right)V_{i}^{T}{\left\{\sum_{i=1}^{n_{+}}V_{i}V_{i}^{T}\right\}}^{-1}\\ & & \times \sum_{i=1}^{n_{+}}\int_{0}^{\tau }\frac{\int_{t}^{\tau }{\hat{S}}_{0}\left(u\right)du}{{\bar{Y}}_{0}\left(t\right)}d{\hat{M}}_{0,i}\left(t\right)V_{i}. \end{array}$$

### 4.2 Mid‐Trial Sample Size Determination

As demonstrated in Section 4.1, specification of the survival and censoring distributions $S_{0}\left(t\right)$ and G(t) can be influential on the calculation of the predicted power for the standard and the augmented tests. Furthermore, as seen in formula (9), the predicted power of the augmented test depends on the variance–covariance matrix of the covariates V and the martingale residuals. It is crucial to accurately specify the quantities in the sample size formula to ensure an accurate power calculation for the *target study*. However, it may be challenging to derive precise estimates for these quantities from the *reference data* that are available at the design stage. With the notable feature that the power formulas (10) and (11) are free from the treatment allocation Z, one can estimate the local power with the mid‐trial blinded data set by pooling the data sets of the two treatment groups. To be specific, we propose to conduct a blind review at an early stage in the *target study* with $n_{mid}$ ($n_{mid}<n$) subjects followed up to τ and then calculate the predicted power for n subjects with estimated ${\tilde{\sigma }}^{2}$ and ${\hat{e}}_{2}$. We can determine the sample size for the statistical analysis with the predicted power to be the target power, say 0.8. Since all the adaptations are made under a blinded review, it would avoid under‐ or overpowered studies, maintaining integrity of the study with the nominal type 1 error rates.

## 5 Simulation Study

### 5.1 Data Generation

We conducted a simulation study investigating the accuracy and effectiveness of the proposed power calculation methods. In this subsection, we explain how to generate three kinds of data sets (*sData 1‐3*). We considered a randomized clinical trial to compare two treatment groups with a time‐to‐event endpoint.

Let $b_{1}$ and $b_{2}$ be independent random variables following the standard normal distribution. We generated two kinds of continuous covariates $V_{1}=b_{1}+\varepsilon_{1}$ and $V_{2}=b_{2}+\varepsilon_{2}$, where $\varepsilon_{1}$ and $\varepsilon_{2}$ followed the standard normal distribution independently. Independence among $b_{1}$, $b_{2}$, $\varepsilon_{1}$, and $\varepsilon_{2}$ was assumed.

To examine the performance of our proposed method, we generated the failure time T, which might be associated with the baseline covariates $V_{1}$ and $V_{2}$ and followed the exponential distribution (*sData 1* and *sData 2*) or the piecewise exponential distribution (*sData 3*) marginally (not conditionally on the baseline covariates). The *sData 1* and *sData 2* were generated from the marginal PH model under the null hypothesis of no treatment effect and the alternative hypothesis, respectively, as follows. The failure time T was generated from the model,

(12) $$\begin{array}{c} \log T=\log\left\{\lambda_{0}\left(1-Z\right)+\lambda_{1}Z\right\}+\log\left(-\log U\right), \end{array}$$

where Z was a binary random variable independent of $V_{1}$ and $V_{2}$, which represented the randomized treatment allocation with P(Z=1)=1/2. U was a random variable, which might or might not be dependent on $V_{1}$ and $V_{2}$ and had the marginal uniform distribution on (0,1). Thus, the failure time distributions for Z=1 and Z=0 were the exponential distribution with the hazard $\lambda_{1}$ and $\lambda_{0}$, respectively. The hazard $\lambda_{0}$ is determined so that the corresponding 5‐year survival rate was 0.2, and $\lambda_{1}$ is determined to satisfy so that the HR $\lambda_{1}/\lambda_{0}$ was 1 (*sData 1*) or 0.7 (*sData 2*). The random variable U was generated under the following two settings; [a] $U=\Phi_{3}\left(b_{1}+b_{2}+\varepsilon \right)$ and [b] $U=\Phi_{1}\left(\varepsilon \right)$, where ε is a standard normal random variable independent of $b_{1}$ and $b_{2}$ and $\Phi_{m}\left(.\right)$ is the cumulative distribution function of the zero‐mean normal distribution with the variance of m. Note that the model (12) is an accelerated failure time model with the error term ε=log(−logU). Since U follows the uniform distribution on (0,1) marginally, ε follows the standard extreme value distribution and then the failure time T follows the exponential distribution. Therefore, both under [a] and [b], the failure time T satisfies the marginal Cox PH model. Under [a], T was dependent on covariates, whereas it was not under [b]. Suppose we are interested in comparing the two groups using the RMST with τ=5. In *sData 1*, there was no treatment effect and then the true RMST difference was 0. In *sData 2*, the true RMST difference was 0.514. The *sData 3* were generated under the non‐PH. For Z=0, the failure time T was generated from the same model as the *sData 1*, the exponential distribution of the hazard $\lambda_{0}$. For Z=1, in a similar way to *sData 1*, T was generated from the piecewise exponential distribution, in which the hazard was $\lambda_{0}$ for t<1 and was a different value $\lambda_{2}$ for 1≤t, so that the resulting true RMST difference was 0.514. The potential censoring time C was generated from the uniform distribution on (0,8). The data set *sData1* with [a] is referred as *sDATA 1a*. Similar notations are used for other combinations. For each combination, 10,000 sets of 500 subjects were generated.

The *sData 1* to *sData 3* were regarded as the *target study*. We generated two kinds of *reference data* for each *sData*. One is from the same distribution of the control group, which is referred as *correctly matched*. The other was from a biased sampling from the control group; subjects of $V_{1}<1$ and $V_{2}<1$ were only sampled, which is referred as *mis‐matched*. Correspondingly to the data sets for the *target study*, 10,000 sets of the *reference data* were generated, each of which included 200 subjects.

### 5.2 Accuracy of the Predicted Power Calculated With the Reference Data at the Design Stage

In Table 1, empirical powers of the standard and augmented RMST tests of n=500 based on 10,000 simulation data sets are presented. Summaries of the predicted power calculated with the *correctly matched* and *mis‐matched* *reference data* are also demonstrated. The empirical sizes were very close to the nominal level of 5% and inclusion of the augmentation term did not lead to inflation of the type 1 error rates. The augmented test had certain gains in power for data sets in which the failure time had dependence of $v_{1}$ and $v_{2}$ (*sData 2a and 3a*). The average of the predicted power with the *correctly matched reference data* (denoted by *cPP*) was close to the corresponding empirical power. On the other hand, those with the *mis‐matched reference data* (*mPP*) were not necessarily close.

**TABLE 1:** Empirical powers of the standard and augmented RMST‐based tests and the average predicted powers calculated at the design stage with the reference data over 10,000 simulated data sets; *Power* means empirical powers, *cPP* and *mPP* are the predicted power with the correctly matched and incorrectly matched *reference data*, respectively.

Data set	Status	Dependence	True	Test	Power	cPP	mPP
sData1a	Null	v1, v2	0	Augmented	0.052	NA	NA
				Standard	0.054	NA	NA
sData1b	Null	None	0	Augmented	0.055	NA	NA
				Standard	0.053	NA	NA
sData2a	PH	v1, v2	0.514	Augmented	0.925	0.939	0.903
				Standard	0.842	0.860	0.873
sData2b	PH	None	0.514	Augmented	0.843	0.863	0.862
				Standard	0.843	0.860	0.860
sData3a	nonPH	v1, v2	0.514	Augmented	0.910	0.906	0.903
				Standard	0.820	0.817	0.872
sData3b	nonPH	None	0.514	Augmented	0.821	0.821	0.863
				Standard	0.819	0.817	0.860

### 5.3 Validity of Adaptive Choice of Sample Size Under a Mid‐Trial Blinded Review of the Target Study

As observed in Section 5.2, if the *reference data* do not reflect the distributional structure of the *target study*, the predicted power might not approximate the power of the *target study* accurately. To the simulation data sets, we applied the proposed mid‐trial sample size evaluation procedure in Section 4.3. At a mid‐trial blinded review with $n_{mid}=100$ or =200, we estimated $S_{0}\left(t\right)$ and G(t) with the pooled data of the two treatment groups. Then, we calculated the predicted power with $n=n_{mid},n_{mid}+10,n_{mid}+20,\text{…}$ and decided the minimum sample size with the predicted power higher than the target power 0.8 as the sample size for the final analysis. Empirical sizes and powers of the blinded adaptive sample size re‐estimation procedure were shown in Table 2 with $n_{mid}=100$ and =200, respectively. The results for *sData 1* indicated that the empirical sizes were close to the nominal level of 0.05 in all the scenarios. From the results for *sData 2* and *sData 3*, the empirical powers were very close to the target power 0.8. Overall, the proposed method successfully controlled the power.

**TABLE 2:** Empirical powers of the standard and augmented RMST‐based tests conducted at the adaptively selected sample size with the predicted power with the earliest $n_{mid}$ subjects under the blind review and summary of sample sizes over 10,000 simulated data sets.

			RMST difference					Adaptively selected sample size				
$n_{mid}$	Test	Status	True	Target	Dependence	Data set	Power	Min	q1	Median	q3	Max
100	Standard	Null	0	0.514	v1, v2	sData1a	0.052	180	390	420	440	640
					None	sData1b	0.055	210	390	420	450	570
		PH	0.514	0.514	v1, v2	sData2a	0.801	200	420	440	470	630
					None	sData2b	0.789	210	420	440	470	610
		Non‐PH	0.514	0.514	v1, v2	sData3a	0.799	190	450	470	500	650
					None	sData3b	0.793	200	450	470	500	630
	Augmented	Null	0	0.514	v1, v2	sData1a	0.056	130	280	310	340	470
					None	sData1b	0.056	190	380	410	440	550
		PH	0.514	0.514	v1, v2	sData2a	0.793	140	310	330	360	480
					None	sData2b	0.781	200	410	430	460	610
		Non‐PH	0.514	0.514	v1, v2	sData3a	0.786	190	440	460	490	630
					None	sData3b	0.794	150	330	360	380	500
200	Standard	Null	0	0.514	v1, v2	sData1a	0.053	280	410	430	450	570
					None	sData1b	0.053	270	410	430	450	540
		PH	0.514	0.514	v1, v2	sData2a	0.814	330	440	450	470	570
					None	sData2b	0.802	330	440	450	470	580
		Non‐PH	0.514	0.514	v1, v2	sData3a	0.810	320	470	480	500	600
					None	sData3b	0.803	380	470	480	500	600
	Augmented	Null	0	0.514	v1, v2	sData1a	0.053	210	300	320	340	450
					None	sData1b	0.055	270	410	430	445	530
		PH	0.514	0.514	v1, v2	sData2a	0.810	230	330	340	360	450
					None	sData2b	0.799	330	430	450	470	570
		Non‐PH	0.514	0.514	v1, v2	sData3a	0.813	250	350	370	390	490
					None	sData3b	0.802	380	460	480	500	590

We also made a similar evaluation for the augmented RMST‐based test with the covariates $V_{1}$ and $V_{2}$. We selected the final sample size of the predicted power 0.8 by the augmented test and the results are summarized in Table 2. It indicated that the empirical sizes were close to the nominal level and the empirical powers were also close to the target one. Thus, these results suggested that the blinded adaptive sample size choice procedure successfully controlled the power maintaining the validity. Table 2 shows the distributions of the sample size selected by the mid‐trial blinded review. With augmentation, the number of sample size might be reduced substantially. Variations of the calculated sample sizes were smaller with $n_{mid}=200$ than with $n_{mid}=100$.

## 6 Examples

### 6.1 Colon Data

In this section, we illustrate our proposing method with a data set from a randomized clinical trial to compare efficacy and safety of the three adjuvant therapies of levamisole alone, levamisole plus fluorouracil (5‐FU), and no therapy (observational group) in resected stage B and C colorectal carcinoma (Laurie et al. 1989; Moertel et al. 1990), which is available as the colon data set in the R package *SURVIVAL*. We pretend to conduct a randomized clinical trial to compare levamisole plus 5‐FU and levamisole alone, which is the *target study*. We regard the data set of the observational group in the *colon* data set as the natural history data set available when designing the *target study* and use it as the *reference data*.

Suppose we compare the overall survival between the levamisole plus 5‐FU group and levamisole alone by using the standard RMST‐based test with the two‐tailed significance level of 0.05. We define τ=1825 (days) and set the minimum clinically important difference as 150 (days) with respect to the RMST‐difference. After excluding subjects with missing values in the covariates listed in Section 6.2, the observational group of the *colon* data contained 305 subjects. It is important to note that while the survival function in the observational group may differ from that in the levamisole‐alone group, we have used the observational group as the *reference data* for illustrative purposes in this example. In practice, it is crucial to select the *reference data* carefully unless the mid‐trial re‐evaluation is conducted. Among the 305 subjects, 164 died. Estimating $S_{0}\left(t\right)$ and G(t) in (7) with the *reference data*, we evaluated the predicted powers with the formula (11). We set the target power 0.8. As presented in Table 3, with n=490, the predicted power was more than 0.8 to detect the RMST‐difference $\theta_{alt}=150$ (days).

**TABLE 3:** Predicted powers of the standard and augmented RMST‐based test with the four selected covariates using the observational group of the colon data as the reference data with two‐tailed 5% significance level and n=490 to detect the true RMST difference of 150 (days). The bolded parts indicate the sample size required to achieve a target power of 0.8 for each method.

	Standard		Augmented	
*n*	Design stage	Blind review	Design stage	Blind review
360	0.676	0.666	0.776	0.711
370	0.688	0.678	0.787	0.723
380	0.700	0.690	0.798	0.734
390	0.711	0.701	**0.808**	0.745
400	0.722	0.712	0.818	0.756
410	0.732	0.722	0.827	0.766
420	0.743	0.733	0.836	0.776
430	0.752	0.743	0.844	0.785
440	0.762	0.752	0.852	0.795
450	0.771	0.761	0.860	**0.803**
460	0.780	0.771	0.867	0.812
470	0.789	0.779	0.874	0.820
480	0.797	0.788	0.881	0.828
490	**0.805**	0.796	0.887	0.836
500	0.813	**0.804**	0.893	0.843

To see how influential the specification of $S_{0}\left(t\right)$ and G(t) is on the calculation of the predicted power, we calculated the predicted power with the exponential distributions for $S_{0}\left(t\right)$ and G(t) with the same 5‐year survival rates as those from the Kaplan–Meier estimates, respectively. The 5‐year survival and censoring probabilities $S_{0}\left(1825\right)$ and G(1825) were estimated as 0.520 and 0.965 with the Kaplan–Meier method. If we assume the exponential distributions for the survival and censoring distributions, the corresponding hazard parameters were $\lambda_{S}=3.58\times 10^{-4}$ and $\lambda_{G}=1.95\times 10^{-5}$, respectively. The predicted power based on these exponential survival and censoring distributions with n=490 was 0.759 to detect the RMST difference of 150 (days), suggesting that inappropriate specification of $S_{0}\left(t\right)$ and G(t) can lead to over‐ or underestimation of the sample size.

Based on the calculation with the *reference data*, we set n=490 as the target sample size. Concerning discrepancy between the *reference data* and the *target study*, we applied the mid‐trial re‐evaluation procedure following the method in Section 4.3. The predicted powers with a randomly selected 200 subjects are shown in Table 3. With n=500, the predicted power attained the target power 0.8.

Next, we determined the target sample size using the augmented RMST‐based test. Moertel et al. (1990) reported several prognosis factors in their Table 1 including extent of local spread (submucosa, muscle, serosa, contiguous structures), the number of lymph *nodes* with detectable cancer, *differentiation* of tumor (well, moderate, poor), *obstruction* of colon by tumor, *perforation* of colon, *adherence* to nearby organs as well as *sex* and *age*. To determine the covariates included in the augmented term, we used a stepwise variable increase method. In the first stage, we considered the augmented RMST‐based test with a single covariate and selected the covariate attaining the maximum ${\hat{e}}_{2}$ over all covariates. In the second stage, we selected the covariate providing the maximum gain in ${\hat{e}}_{2}$ by adding to the covariate selected in the first stage. This step was continued until all covariates were included in the model. In Table 4, the covariates selected in the process and the predicted power with n=490 are shown. In the first stage, *nodes* had the maximum gain in ${\hat{e}}_{2}$. In the second stage, we evaluated gains in ${\hat{e}}_{2}$ by adding one more covariate to *nodes*, and selected *differentiation*. Table 4 indicates that improvement in power was saturated at stage 3; the predicted power with the set of covariates of *nodes*, *differentiation*, and *extent* was almost the same as that with all eight covariates. As seen in Equation (9), the inverse of $E(VV^{T})$ must be taken to calculate the predicted power. Thus, unnecessary variables should not be included in the augmented term to avoid a colinearity problem. In the present case, we selected three covariates.

**TABLE 4:** Predicted powers of the standard and augmented RMST‐based tests with the colon data as the *reference data*; # implies the number of covariates included in the augmented term, Power is the predicted power with n=490, which has the power of 0.8 for the standard RMST‐based test. Variables indicates the covariates of maximum gain in power by adding sequentially. For example, in the augmented logrank test with a single covariate, *nodes* had the maximum value of ${\hat{e}}_{2}$ and *+differentiation* implies *differentiation* gave the maximum gain in the value of ${\hat{e}}_{2}$ by adding a single covariate to *nodes*.

Step	Variables	$e_{2}$	Power
0			0.805
1	*+differentiation*	228207.8	0.821
2	*+nodes*	865255.6	0.867
3	*+local*	1136876	0.886
4	*+sex*	1159761	0.887
5	*+obstruction*	1164897	0.888
6	*+perforation*	1166504	0.888
7	*+age*	1166505	0.888
8	*+adherence*	1166509	0.888

In Table 3, the predicted powers of the augmented test with the three covariates based on the *reference data* are shown; with n=390, the predicted power attained the target power of 0.8. The results of the mid‐trial re‐evaluation at $n_{mid}=200$ are also shown in Table 3, indicating that n=450 attains the target power of 0.8 and is suggested as the sample size with which the final statistical analysis is conducted. In this example, we observed that the recommended sample size with the mid‐trial blinded evaluation might be so different from that with the *reference data* for the augmented test. We compared the RMSTs of the two groups with n=450 subjects and summarized the results of the estimation in Table 5. The standard estimate of the RMST difference was 131.6 (95% confidence interval (CI): 25.1, 239.2) days. The augmented one with the selected three covariates gave the estimate 135.3 (95% CI: 32.4, 238.2). The addition of the augmentation term made the length of the confidence interval certainly shorter. The augmented estimate with all eight covariates had a similar standard error to that with the selected three covariates as suggested in Table 4.

**TABLE 5:** Results of comparison of the RMST difference of the two treatments in the *colon data* with n=450 based on the standard and augmented tests; “augmented (selected)” and “augmented (all)” imply the augmented test with the three selected covariates and that with all the eight covariates, respectively.

Method	RMST difference (SE)	95%CI	p‐value
Standard	131.6 (54.9)	(25.1, 239.2)	0.016
Augmented (selected)	135.3 (52.5)	(32.4, 238.2)	<0.001
Augmented (all)	134.5 (52.2)	(32.3, 236.8)	<0.001

### 6.2 The Oak Study

The Poplar study is an open‐label Phase 2 study to compare the efficacy and safety of atezolizumab with docetaxel for non‐small cell lung cancer (Fehrenbacher et al. 2016). Two hundred and eighty seven subjects were enrolled and were randomly assigned to one of the two treatments. The primary endpoint was overall survival and the HR of atezolizumab to docetaxel was estimated as 0.73 (95% CI: 0.53, 0.99; *p* = 0.040). It was followed by the Oak study, which was a large‐scale randomized confirmatory Phase 3 study to compare atezolizumab with docetaxel for non‐small cell lung cancer (Rittmeyer et al. 2017). Eighty hundred and fifty patients were randomized to one of the two treatments. The HR of atezolizumab to docetaxel was estimated as 0.73 (95% CI: 0.62, 0.87; *p* <0.001). As seen in fig. 3A for the Poplar study (Fehrenbacher et al. 2016) and fig. 2A for the Oak study (Rittmeyer et al. 2017), there were delayed responses of immunotherapy observed and then the PH assumption did not seem to hold. We used these two studies (Fehrenbacher et al. 2016; Rittmeyer et al. 2017) for illustrating our proposed methods.

The RMST difference with τ=18 (months) was estimated as 1.22 (−0.23, 2.67) with *p* = 0.099 in the Poplar study. We created an example study using the Oak study and the Poplar study. We regarded the Oak study as the *target study*, and the Poplar study as the *reference data*. Here, we used the control group in the Poplar study as the *reference data*. We set the RMST difference with τ=18 (months) as the treatment contrast and set 1.5 months as the target RMST difference. We begin with the standard RMST test. By estimating $S_{0}\left(t\right)$ and G(t) with the *reference data*, the sample size attaining 1−β=0.9 with a two‐tailed 5% significance level was calculated as 710. We re‐evaluated the sample size with the mid‐trial blinded sample size re‐estimation of the Oak study, in which randomly selected $n_{mid}=200$ subjects were used. With the estimates of $S_{0}\left(t\right)$ and G(t) at the mid‐trial re‐estimation, the sample size was calculated as 860. The Poplar study (Phase 2) was conducted under a similar study protocol to the Oak study (Phase 3); in both studies, the control treatment was docetaxel, the primary endpoint was the overall survival, and the treatments were continued until disease progression. Thus, use of the Poplar study as the *reference data* would be relevant. On the other hand, this may not be typical; Phase 2 studies often have a shorter follow‐up duration than the Phase 3 study. If this is the case, one should be careful about whether the survival function and the censoring distribution up to the truncation time τ, which is used in the target study, can be well estimated with the *reference data*.

Next, we considered the augmented test for the RMST difference as the primary analysis and evaluate the power. We used eight covariates for augmentation; the number of metastatic site (*metastasis*), age at baseline (*age*), smoking status (current, previous and never) (*smoke*), sex (*sex*), histology (Non‐small cell lung cancer, Squamous cell cartinoma) (*histology*), race (White, Asian, others) (*race*), ECOG performance status (0 or 1) (*egoggr*), baseline sum of the longest diameters (*blSLD*), and the number of prior chemotherapies (1 or 2) (*priortrt*). We applied the stepwise variable increase method introduced in Section 6.1 to select the variables included in the augmented term. The history of the selection is presented in Table 6, in which the variable selected at each stage and the predicted power with *n* = 710 are presented. The predicted power seemed to be saturated at step 5. Then, we selected the five variables of *metastasis*, *age*, *smoke*, *sex*, and *histology*. When we included these, the predicted power was 0.947. The number of subjects attaining the target power 0.9 was calculated as 580 with the five covariates. The augmentation could reduce the number of subjects substantially. We re‐evaluated the predicted power at the blinded review with the 200 subjects in the Oak study. The sample size assuring the target power 0.9 was calculated as 750.

**TABLE 6:** Predicted powers of the standard and augmented RMST‐based tests with the Poplar study data as the *reference data*; # implies the number of covariates included in the augmented term, PP is the predicted power with n=710, which has the power of 0.9 for the standard RMST‐based test. Variables indicates the covariates of maximum gain in power by adding sequentially. For example, in the augmented logrank test with a single covariate, *nodes* had the maximum value of ${\hat{e}}_{2}$ and *+differentiation* implies *differentiation* gave the maximum gain in the value of ${\hat{e}}_{2}$ by adding a single covariate to *nodes*.

Step	Variable	$e_{2}$	Power
0			0.900
1	*+metastasis*	15.259	0.907
2	*+age*	72.743	0.933
3	*+smoke*	91.191	0.940
4	*+sex*	103.727	0.945
5	*+histology*	109.419	0.947
6	*+race*	114.078	0.949
7	*+blSLD*	115.638	0.950
8	*+ecogger*	116.686	0.950
9	*+prioritrt*	116.686	0.950

The Poplar and the Oak studies shared many inclusion criteria. However, there was a substantial difference between the predicted power calculation at the design stage with the Poplar study data and the mid‐trial blinded sample size re‐estimation with the Oak study data. The latter only allowed to enroll stage IIIB and IV patients. It might be influential on the association between covariates and the overall survival. We applied a regression model for the RMST difference with the inverse probability censoring weighted method (Tian et al. 2014). We observed that histology was significantly associated with the overall survival in the Oak study, but not in the Poplar study. Such a difference of prognosis between the studies might affect the predicted power calculation. In Table 7, we show the RMST differences estimated with n=580 or n=750 subjects. With n=580, significance was marginal and the mid‐trial sample size recalculation seemed to successfully adjust the sample size. 

**TABLE 7:** Results of comparison of the RMST difference of the two treatments in the *Oak data* based on the standard and augmented tests with n=580, which was determined with the *reference data* and with n=750, which was determined with the mid‐trial sample size re‐estimation.

*n*	Method	RMST difference (SE)	95%CI	p‐value
580	Standard	0.985 (0537)	(−0.067, 2.038)	0.066
	Augmented	1.052 (0.511)	(0.050, 2.053)	0.040
750	Standard	1.107 (0.466)	(0.193, 2.021)	0.018
	Augmented	1.102 (0.448)	(0.225, 1.979)	0.014

## 7 Discussion

In randomized clinical trials with a time‐to‐event endpoint, the logrank‐HR approach is routinely used. An advantage of this strategy is applicability of the event‐driven study design, where the final analysis is conducted when the number of observed events from the study reaches the target. This approach achieves the target power to detect a given HR, if the PH assumption is correct. Whether the survival functions of both groups are correctly specified or not does not affect the power of the final analysis by logrank test or HR‐based tests. On the other hand, the power of the conventional RMST‐based test may be under‐ or overestimated if the survival functions are not correctly specified at the design stage. This may be a challenge when it is used for confirmatory clinical trials (Yuan et al. 2020).

In this paper, we used a local power formula for the RMST‐based test and proposed a method to determine the timing of the final analysis, which resulted in the target power for detecting the target effect size. Our method is based on the idea of the blinded sample size calculation, which is one of the most accurate adaptive design techniques with minimal risk of violation of study integrity (FDA Guidance for Industry 2016;2019). The success of our method was based on the fact that the local power does not depend on the treatment allocation once the target treatment effect in terms of the RMST difference is fixed. There may be a concern that the local power formula does not provide an accurate approximation to the power when the target treatment effect is large. If the target treatment effect is large, the required sample size would be very small. In general, the proposed method is recommended to apply when the number of subjects is not too small. Nevertheless, we contend that this issue does not pose a significant concern within our methodology, as blinded sample‐size re‐estimation is typically implemented with a sufficiently large number of subjects. The proposed method would eliminate a drawback of the conventional RMST‐based design and might make it more feasible to design confirmatory studies with the RMST.

We demonstrated two applications. In the first example of the colon data, the two chemotherapy groups were regarded as comparative groups of the *target study* and the *reference data* was artificially created from the observational arm of the same study. Since the *reference data* was one of the randomized arms in reality, distributions of covariates should be similar. Despite this, the predicted power with the *reference data* was not necessarily close to the predicted power with the mid‐trial sample size recalculation. The situation in the second example can occur frequently; Phase 3 studies are designed with results of Phase 2 studies with similar inclusion criteria. We observed a substantial difference between the predicted power at the design stage calculated with the Phase 2 study and the mid‐trial blinded sample size recalculation. These inconsistencies might happen due to inconsistencies of the associations between covariates and overall survival. If the associations between the failure time and the covariates in the target study are much weaker than those expected at the design stage, the sample size calculated at the design stage can be too optimistic, resulting in underpowered studies. Inconsistency in the survival functions or the censoring distributions can also occur in practice and result in inaccurate sample size calculation at the design stage. For example, the *reference data* may have a shorter follow‐up duration than the *target study* resulting in inappropriate estimates for the survival function and the censoring distribution and then inadequacy of the *reference data*. Thus, careful consideration on the choice of the *reference data* is required at the design stage and re‐evaluation of the sample size with updated predicted powers at mid‐trial blinded reviews is highly recommended to adjust potentially inappropriate estimates at the design stage and assure the target power for the target treatment effect.

The key idea of the proposed method was to estimate the local power with blinded data, which was called the predicted power. Recently, Hattori et al. (2022) proposed a method to determine the number of subjects to conduct a testing hypothesis with the augmented version of the logrank test. The predicted power was monitored and the analysis was conducted at the date when the predicted power attained the target power. For the augmented logrank test, the predicted power increases as the number of events does over time. On the other hand, for the RMST‐based tests, the predicted power is not always the case since it uses only information up to the truncation time τ. Therefore, it would not be appropriate to monitor the timing of analysis based on the predicted power for RMST. Instead, the predicted power should be evaluated at an interim look of blinded data. We should be careful in the choice of the number of subjects $n_{mid}$ for the blinded review accounting for the truncation time and the rate of enrollment of subjects; if the truncation time is long and the rate of enrollment is high, all the subjects determined at the design stage may have already been enrolled at the blinded review and if this is the case, it may be challenging to increase the sample size. To obtain stable recalculated sample sizes, rather larger $n_{mid}$ would be better. Thus, the choice of $n_{mid}$ should be determined on the balance between the stability of the recalculation and feasibility of increasing sample size. Establishing more concrete guidance would be an important research topic.

As argued, we assume that the truncation time τ is fixed and prespecified in the protocol. We believe that in confirmatory comparative clinical trials, the truncation time should be prespecified. On the other hand, data‐dependent choice of τ is discussed by some papers (Horiguchi et al. 2018; Zhao et al. 2016). Our method cannot be applied to this case and it would be warranted to examine how to ensure the target power for the target RMST difference with a data‐dependent truncation time.

In confirmatory clinical trials, interim analysis is widely used to consider early establishment of efficacy and early stopping of the study. For the RMST, Lu and Tian (2021) discussed the group sequential interim analysis methodology. Its extension to the augmented version would be beneficial in practice with potential reduction of sample size. Our current development is limited to the blinded consideration. Further research is warranted on using the proposed method in combination with unblinded interim analysis methodology.

## Conflicts of Interest

The authors have declared no conflicts of interest.

### Open Research Badges

This article has earned an Open Data badge for making publicly available the digitally‐shareable data necessary to reproduce the reported results. The data is available in the Supporting Information section.

This article has earned an open data badge “**Reproducible Research**” for making publicly available the code necessary to reproduce the reported results. The results reported in this article could fully be reproduced.

## Data Availability

The program codes and data sets for the simulation study in Section 5 and the illustration in Section 6 are available on zenodo with the id 10829335 (DOI: 10.5281/zenodo.10829335).

## Appendix A Consistency of ${\hat{\sigma }}_{1}^{2}$ and ${\hat{\sigma }}_{2}^{2}$

From the martingale representation of the Kaplan–Meier estimator, it holds that

$$\begin{array}{ccc} \sqrt{n_{z}}\left\{{\hat{S}}_{z}\left(t\right)-S_{z}\left(t\right)\right\} & = & -S_{z}\left(t\right)\sqrt{n_{z}}n^{-1}\sum_{i=1}^{n}\int_{0}^{t}\frac{dM_{z,i}\left(u\right)}{{\bar{Y}}_{z}\left(u\right)}+o_{p}\left(1\right), \end{array}$$

where ${\bar{Y}}_{1}\left(u\right)=n^{-1}\sum_{i=1}^{n}I\left(X_{i}\geq u\right)Z_{i}$ and ${\bar{Y}}_{0}\left(u\right)=n^{-1}\sum_{i=1}^{n}I\left(X_{i}\geq u\right)\left(1-Z_{i}\right)$. With this representation, simple algebraic manipulation entails that

(A1)
$$\begin{array}{ccc} \sqrt{n}\left({\hat{\theta }}_{1}-\theta_{1}\right) & = & \frac{\sqrt{n}}{\sqrt{n_{1}}}\int_{0}^{\tau }\sqrt{n_{1}}\left\{{\hat{S}}_{1}\left(t\right)-S_{1}\left(t\right)\right\}ds\\ & ≃ & -\frac{1}{\sqrt{n}}\sum_{i=1}^{n}Z_{i}\int_{0}^{\tau }\frac{\int_{u}^{\tau }S_{1}\left(s\right)ds}{E\{I\left(X_{i}\geq u\right)Z_{i}\}}dM_{1,i}\left(u\right). \end{array}$$

Similarly,

(A2)
$$\begin{array}{ccc} \sqrt{n}\left\{{\hat{\theta }}_{0}-\theta_{0}\right\} & ≃ & -\frac{1}{\sqrt{n}}\sum_{i=1}^{n}\left(1-Z_{i}\right)\int_{0}^{\tau }\frac{\int_{u}^{\tau }S_{0}\left(s\right)ds}{E\{I\left(X_{i}\geq u\right)\left(1-Z_{i}\right)\}}dM_{0,i}\left(u\right). \end{array}$$

The standard moment calculus of the counting process martingale (Fleming and Harrington 1991) derives the asymptotic variance $\sigma^{2}$ and the consistency of ${\hat{\sigma }}_{2}^{2}$.

## Appendix B Derivation of $\hat{c}$ and Consistency of ${\hat{\sigma }}_{aug}^{2}$

From (A1) and (A2),

$$\begin{array}{cc} & \sqrt{n}\left\{{\hat{\theta }}_{aug}\left(c\right)-\theta \right\}=\sqrt{n}\left\{\hat{\theta }-AUG\left(c\right)-\theta \right\}\\ & \;≃\frac{1}{\sqrt{n}}\sum_{i=1}^{n}[\int_{0}^{\tau }\{\frac{-Z_{i}\int_{u}^{\tau }S_{1}\left(s\right)ds}{E\{I\left(X_{i}\geq u\right)Z_{i}\}}dM_{1,i}\left(u\right)\\ & \;+\int_{0}^{\tau }\frac{\left(1-Z_{i}\right)\int_{u}^{\tau }S_{0}\left(s\right)ds}{E\{I\left(X_{i}\geq u\right)\left(1-Z_{i}\right)\}}dM_{0,i}\left(u\right)\}\\ & \;-c^{T}\left(Z_{i}-\pi \right)V_{i}]. \end{array}$$

Then, the variance of $\sqrt{(}n)\left({\hat{\theta }}_{aug}\left(c\right)-\theta \right)$ converges to

$$\begin{array}{cc} & E[\int_{0}^{\tau }\{\frac{-Z\int_{u}^{\tau }S_{1}\left(s\right)ds}{E\{I(X\geq u)Z\}}dM_{1}\left(u\right)+\frac{\left(1-Z\right)\int_{u}^{\tau }S_{0}\left(s\right)ds}{E\{I(X\geq u)(1-Z)\}}dM_{0}\left(u\right)\}\\ & \;{-c^{T}\left(Z-\pi \right)V]}^{2}. \end{array}$$

A simple algebraic manipulation gives us the minimizer as

$$\begin{array}{cc} c^{*} & ={\left[E\left\{{\left(Z-\pi \right)}^{2}VV^{T}\right\}\right]}^{-1}\\ & \;\times E[\left(Z-\pi \right)V\{\frac{-Z\int_{u}^{\tau }S_{1}\left(s\right)ds}{E\{I(X\geq u)Z\}}dM_{1}\left(u\right)\\ & \;+\int_{0}^{\tau }\frac{\left(1-Z\right)\int_{u}^{\tau }S_{0}\left(s\right)ds}{E\{I(X\geq u)(1-Z)\}}dM_{0}\left(u\right)\}], \end{array}$$

which is consistently estimated by $\hat{c}$ from the standard law of large number. It holds that $n^{-\frac{1}{2}}\sum_{i=1}^{n}\left(Z_{i}-\pi \right){\hat{c}}^{T}V_{i}=n^{-\frac{1}{2}}\sum_{i=1}^{n}\left(Z_{i}-\pi \right)c_{*}^{T}V_{i}+o_{p}\left(1\right)$. Then, by the standard central limit theorem, the asymptotic normality of $\sqrt{n}\left({\hat{\theta }}_{aug}-\theta \right)$ and the consistency of ${\hat{\sigma }}_{aug}^{2}$ holds.

## Appendix C Derivation of (7)

From (5), it holds that

(C1)
$$\begin{array}{ccc} \sqrt{n}AUG\left(\hat{c}\right) & = & {\hat{c}}^{T}\frac{1}{\sqrt{n}}\sum_{i=1}^{n}\left(Z_{i}-\pi \right)V_{i} \end{array}$$

(C2)
$$\begin{array}{ccc} & = & \frac{1}{n}\sum_{i=1}^{n}\left(Z_{i}-\pi \right)V_{i}\left[-Z_{i}\int_{0}^{\tau }\frac{\int_{u}^{\tau }S_{1}\left(s\right)ds}{E\{I\left(X_{i}\geq u\right)Z_{i}\}}dM_{1,i}\left(u\right)\right.\\ & & \left.+\left(1-Z_{i}\right)\int_{0}^{\tau }\frac{\int_{u}^{\tau }S_{0}\left(s\right)ds}{E\{I\left(X_{i}\geq u\right)\left(1-Z_{i}\right)\}}dM_{0,i}\left(u\right)\right] \end{array}$$

(C3)
$$\begin{array}{ccc} & \times & {\left\{\pi \left(1-\pi \right)\frac{1}{n}\sum_{i=1}^{n}V_{i}V_{i}^{T}\right\}}^{-1}\frac{1}{\sqrt{n}}\sum_{i=1}^{n}\left(Z_{i}-\pi \right)V_{i}. \end{array}$$

By simple algebra, (C2) equals to

(C4)
$$\begin{array}{ccc} & & -\frac{1}{n}\sum_{i=1}^{n}{\left(Z_{i}-\pi \right)}^{2}V_{i}^{T}\int_{0}^{\tau }[\frac{\int_{u}^{\tau }S_{1}\left(s\right)ds}{E\{I\left(X_{i}\geq u\right)Z_{i}\}}dM_{1,i}\left(u\right)\\ & & +\frac{\int_{u}^{\tau }S_{0}\left(s\right)ds}{E\{I\left(X_{i}\geq u\right)\left(1-Z_{i}\right)\}}dM_{0,i}\left(u\right)]\\ & & -\pi \frac{1}{n}\sum_{i=1}^{n}\left(Z_{i}-\pi \right)V_{i}^{T}\int_{0}^{\tau }\frac{\int_{u}^{\tau }S_{1}\left(s\right)ds}{E\{I\left(X_{i}\geq u\right)Z_{i}\}}dM_{1,i}\left(u\right) \end{array}$$

(C5)
$$\begin{array}{c} +\left(1-\pi \right)\frac{1}{n}\sum_{i=1}^{n}\left(Z_{i}-\pi \right)V_{i}^{T}\int_{0}^{\tau }\frac{\int_{u}^{\tau }S_{0}\left(s\right)ds}{E\{I\left(X_{i}\geq u\right)\left(1-Z_{i}\right)\}}dM_{0,i}\left(u\right). \end{array}$$

As argued in the Section 3.2, $S_{1}\left(t\right)=S_{0}\left(t\right)+o\left(1\right)$, $\Lambda_{1}\left(t\right)=\Lambda_{0}\left(t\right)+o\left(1\right)$, $M_{1,i}\left(t\right)=M_{0,i}\left(t\right)+o_{p}\left(1\right)$, $E\left\{I\left(X\geq t\right)Z\right\}=S_{1}\left(t\right)G\left(t\right)\pi =S_{0}\left(t\right)G\left(t\right)\pi =o\left(1\right)$, and $E\left\{I\left(X\geq t\right)\left(1-Z\right)\right\}=S_{0}\left(t\right)G\left(t\right)\left(1-\pi \right)$. With these relationships,

$$\begin{array}{ccc} \left(C4\right) & ≃ & -\frac{1}{n}\sum_{i=1}^{n}\left(Z_{i}-\pi \right)V_{i}^{T}\int_{0}^{\tau }\frac{\int_{u}^{\tau }S_{0}\left(s\right)ds}{S_{0}\left(u\right)G\left(u\right)}dM_{0,i}\left(u\right) \end{array}$$

and

$$\begin{array}{ccc} \left(C5\right) & ≃ & \frac{1}{n}\sum_{i=1}^{n}\left(Z_{i}-\pi \right)V_{i}^{T}\int_{0}^{\tau }\frac{\int_{u}^{\tau }S_{0}\left(s\right)ds}{S_{0}\left(u\right)G\left(u\right)}dM_{0,i}\left(u\right), \end{array}$$

and thus (C4) plus (C5) is $o_{p}\left(1\right)$. Then, it holds

(C6)
$$\begin{array}{ccc} \left(C2\right) & ≃ & -\frac{1}{n}\sum_{i=1}^{n}{\left(Z_{i}-\pi \right)}^{2}V_{i}^{T}\int_{0}^{\tau }\left[\frac{1}{\pi (1-\pi )}\frac{\int_{u}^{\tau }S_{0}\left(s\right)ds}{S_{0}\left(u\right)G\left(u\right)}dM_{0,i}\left(u\right)\right]\\ & ≃ & -\frac{1}{\pi (1-\pi )}E\left[{\left(Z-\pi \right)}^{2}\int_{0}^{\tau }\frac{\int_{u}^{\tau }S_{0}\left(s\right)ds}{S_{0}\left(u\right)G\left(u\right)}dM_{0}\left(u\right)V^{T}\right]\\ & = & -E\left[\int_{0}^{\tau }\frac{\int_{u}^{\tau }S_{0}\left(s\right)ds}{S_{0}\left(u\right)G\left(u\right)}dM_{0}\left(u\right)V^{T}\right] \end{array}$$

where the last equality holds since ${\left(Z_{i}-\pi \right)}^{2}=1/4=\pi \left(1-\pi \right)$ algebraically when π=1/2. In (C3), $n^{-1}\sum_{i=1}^{n}V_{i}V_{i}^{T}≃E\left(VV^{T}\right)$ and from Condition 1

$$\begin{array}{c} Var\left(\frac{1}{\sqrt{n}}\sum_{i=1}^{n}\left(Z_{i}-\pi \right)V_{i}\right)≃\pi \left(1-\pi \right)E\left(VV^{T}\right). \end{array}$$

Then, $Var(\sqrt{n}AUG_{2})$ asymptotically agrees with (8).
